# Supplementary material for: Double trouble: two retrotransposons triggered a cascade of invasions in Drosophila species within the last 50 years
Source: Nat Commun. 2025 Jan 9;16:516. doi: 10.1038/s41467-024-55779-6 (PMC11718211; doi:10.1038/s41467-024-55779-6)
Supplement: Supplementary file 1 — Supplementary Material [file 41467_2024_55779_MOESM1_ESM.pdf]

Supplementary figures and tables

Supplementary figures

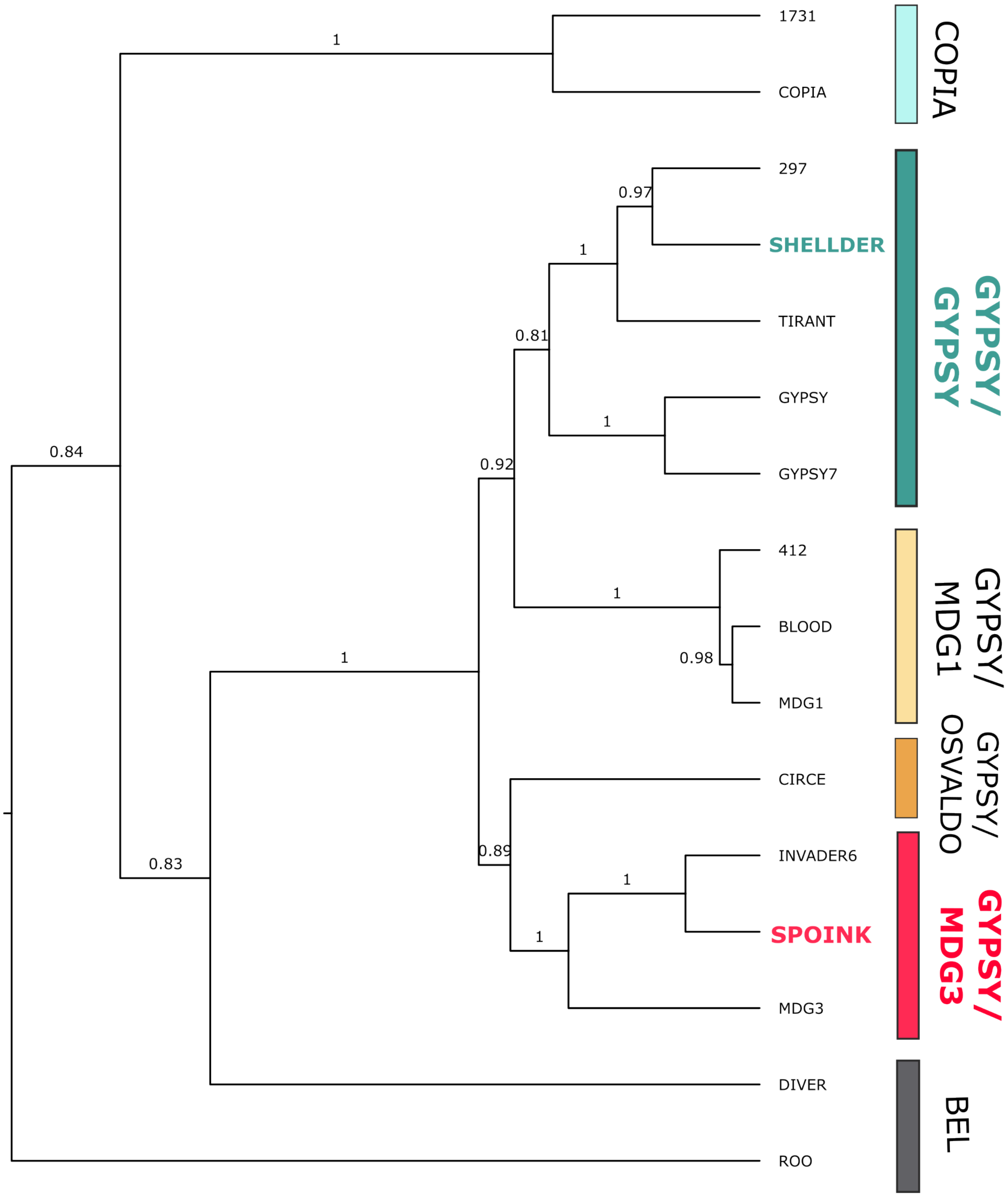

Figure 1: Phylogenetic tree based on the reverse-transcriptase domain of *pol* for *Spoink*, *Shellder* and several other LTR transposons. Multiple families have been picked for each of the main superfamilies/groups of LTR transposons [Kapitonov and Jurka, 2003]. As reported previously *Spoink* is a member of the the gypsy/mdg3 group [Pianezza et al., 2023]. Our data further suggest that *Shellder* is a member of the gypsy/gypsy group.

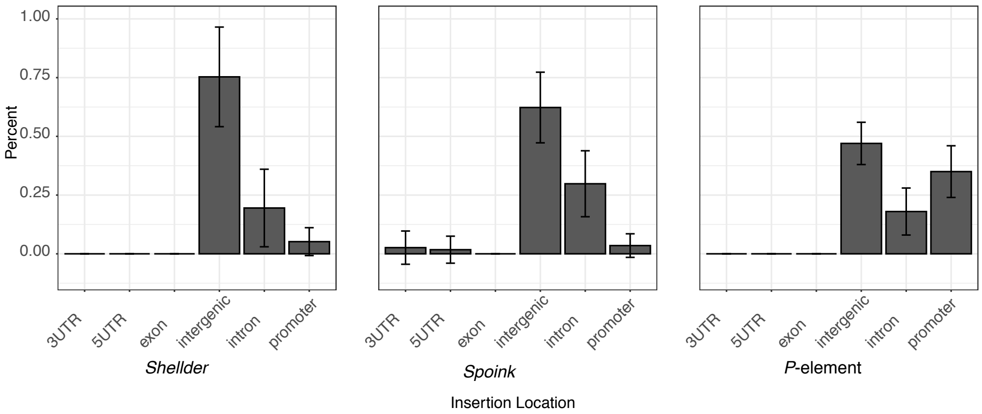

Figure 2: Summary of insertion locations for *Shellder*, *Spoink*, and the *P-element* in *D. simulans*. TE insertions were identified in 10 long-read assemblies of *D. simulans* [Signor et al., 2023] and the reference annotation was lifted to each assembly with liftoff [Shumate and Salzberg, 2021, Gramates et al., 2022]. The *P-element* was only documented in three assemblies as most of these strains were collected prior to its invasion. Note that we previously reported that *Spoink* insertions are largely found in introns and intergenic regions whereas the *P-element* has a pronounced insertion bias in promoters (defined as 1000bp upstream of the first exon) [Pianezza et al., 2023] *Shellder* has a pronounced insertion bias into intergenic regions.

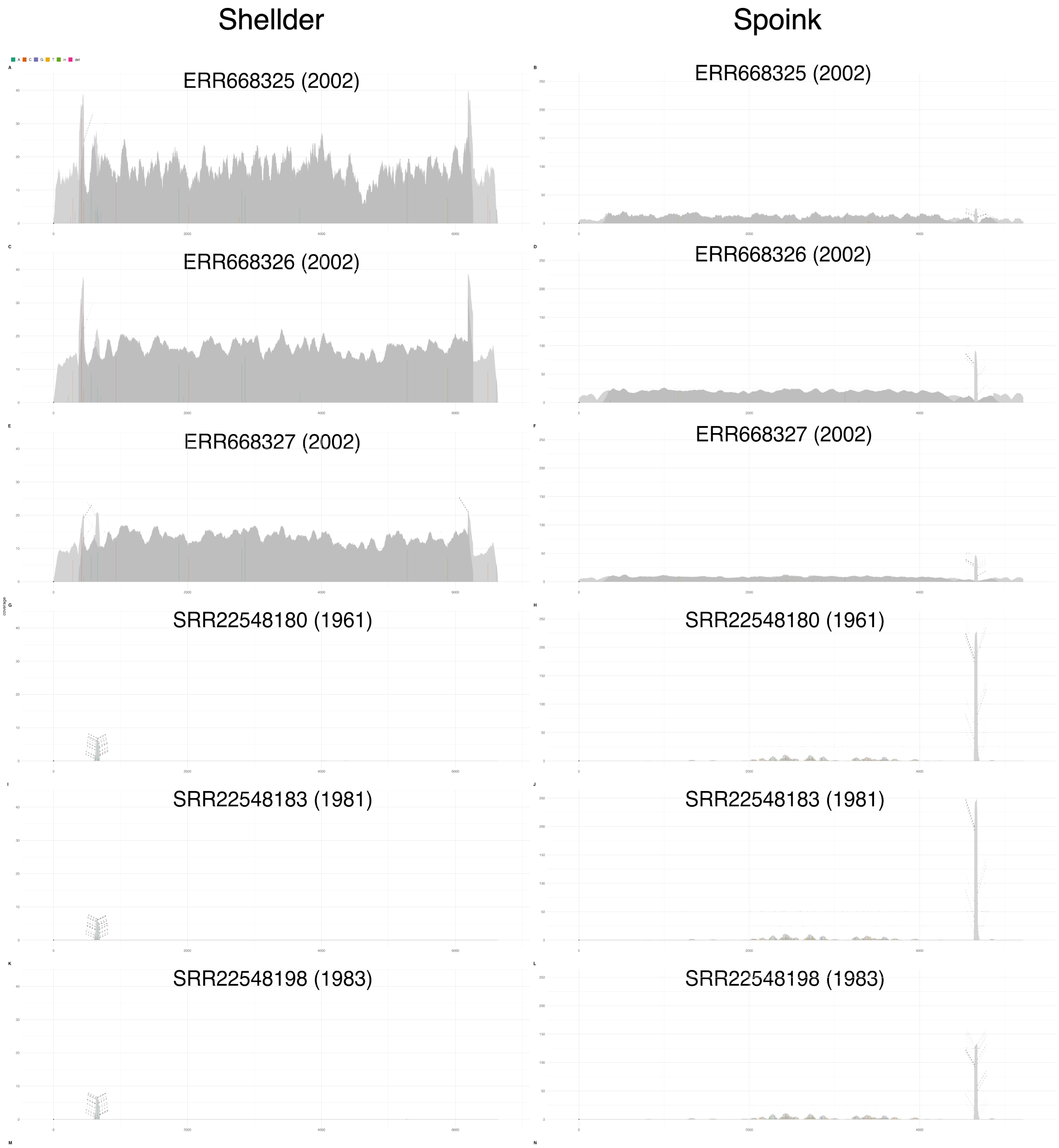

Figure 3: DeviaTE plots of six *D. simulans* strains collected during the last century. The short reads were aligned to the consensus sequence of *Spoink* and the coverage was normalized to the the coverage of single-copy genes. Note that very few reads of old strains ($\leq$ 1975) align to *Spoink* and *Shellder* whereas a contiguous coverage of reads along both TEs is observed for more recently collected strains ($\geq$ 1993). The coverage peak towards the 3' end *Spoink* is due to a poly-A track [Pianezza et al., 2023].

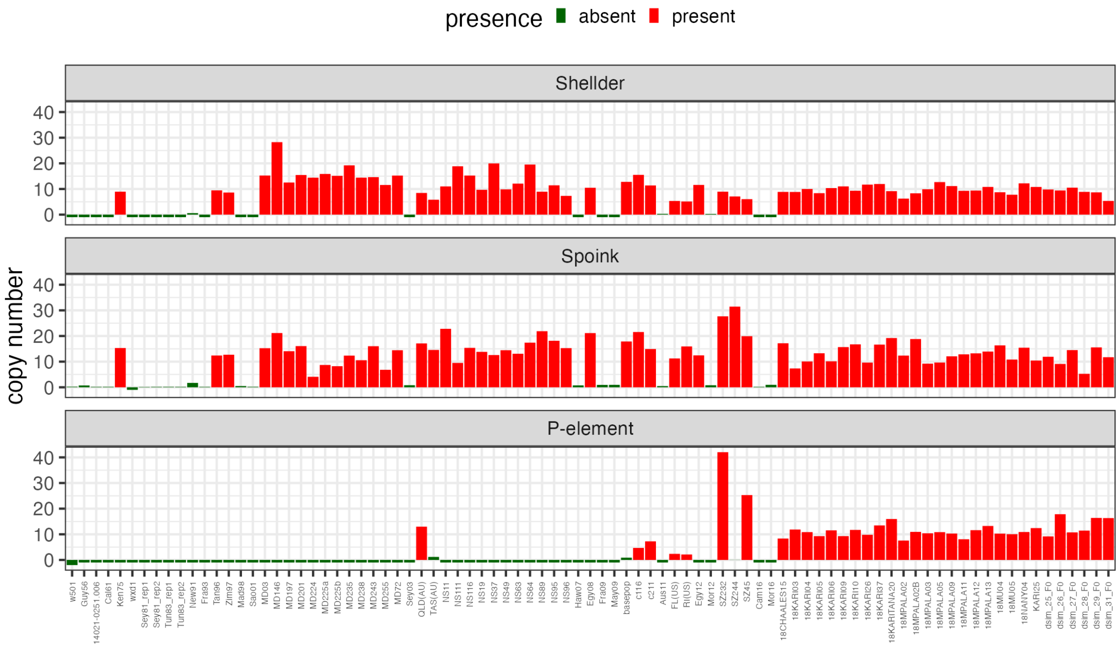

Figure 4: Abundance of *Spoink*, *Shellder* and the *P-element* in 88 *D. simulans* (data were obtained via short-read sequencing). Strains were collected at different geographic regions during the last 70 years. Note that *Spoink* and *Shellder* are either both present or absent in a given strain. Strains are ordered by collection year.

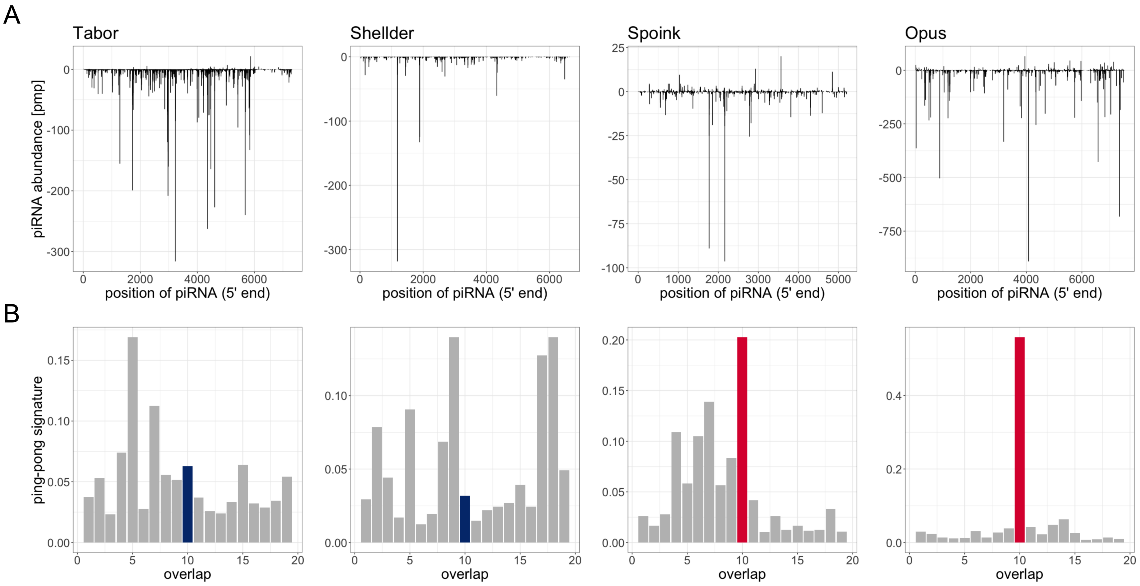

Figure 5: In *D. simulans*, *Spoink* is silenced by the germline piRNA pathway and *Shellder* by the somatic pathway. As references we included a typical somatic (Tabor) and germline TE (Opus). A) Distribution of piRNAs along the sequence of the TEs. Note that *Shellder* only has antisense piRNAs whereas sense and antisense piRNAs can be found *Spoink*. B) Ping-pong signature for the TEs. *Spoink* has a notable ping-pong siganture (red) whereas *Shellder* does not (blue).

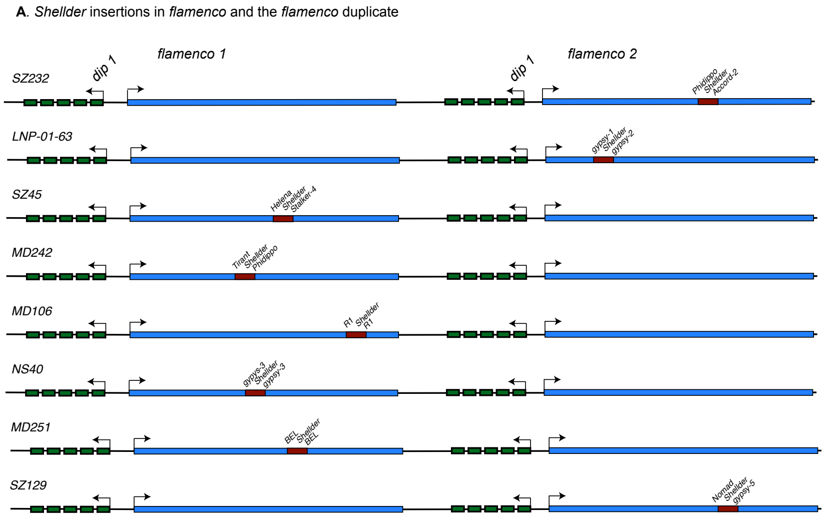

Figure 6: Insertions of *Shellder* into *flamenco* in long-read assemblies of *D. simulans*. Locations are approximate, neighboring TEs are shown to illustrate the lack of synteny in insertion site. Some strains do not have complete assemblies of *flamenco*, however if there was a *Shellder* insertion in the assembled portion it is shown here. For the following reasons we propose that each *Shellder* insertion into *flamenco* is due to a separate insertion event. First, the insertions are at different positions along the *flamenco* locus. Second, these insertions are not flanked by the same TEs, thus it is not a case of rearrangement of the locus unless the breakpoints were precisely neighboring the *Shellder* insertion. Lastly, three of the insertions are into the *flamenco* duplicate rather than the original *flamenco*.

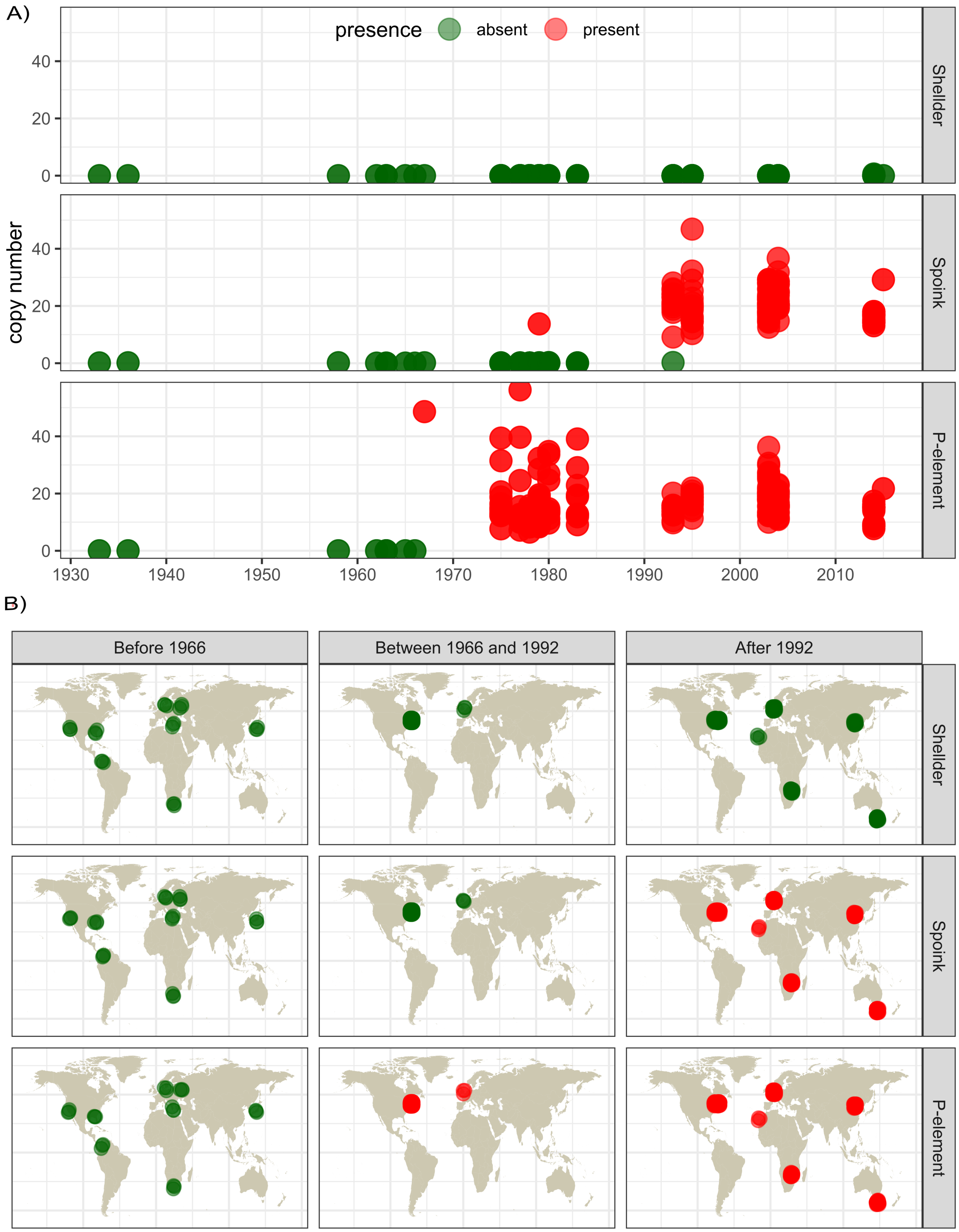

Figure 7: While *Spoink* and the *P-element* recently invaded worldwide *D. melanogaster* populations, *Shellder* is absent in all analysed strains. A) Abundance of *Spoink*, *Shellder* and *P-element* in different *D. melanogaster* strains collected during the last 100 years. B) Geographic spread of *Spoink*, *Shellder*, and the *P-element* in worldwide *D. melanogaster* populations during the last decades. Strains having a given TE are shown in red while strains not having the TE are green.

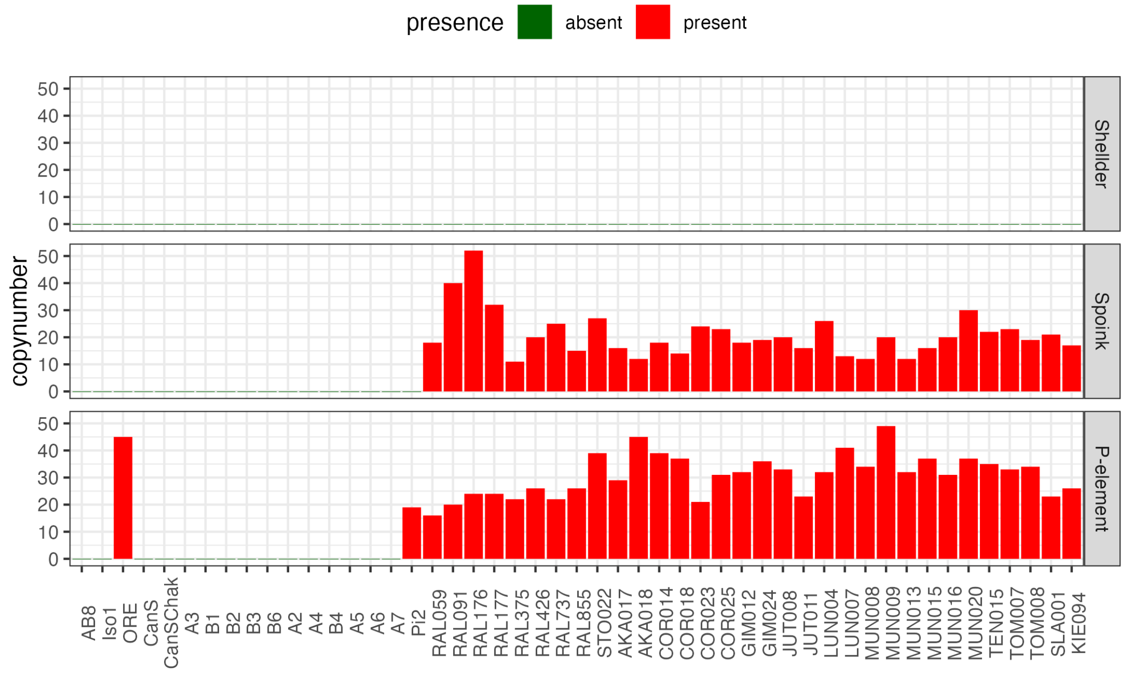

Figure 8: Abundance of *Shellder*, *Spoink* and *P-element* insertions in 49 long reads assemblies of different *D. melanogaster* strains. Insertions were identified with RepeatMasker and hits with less than 750bp length or more than 10% divergence were removed. Strains are ordered by collection year.

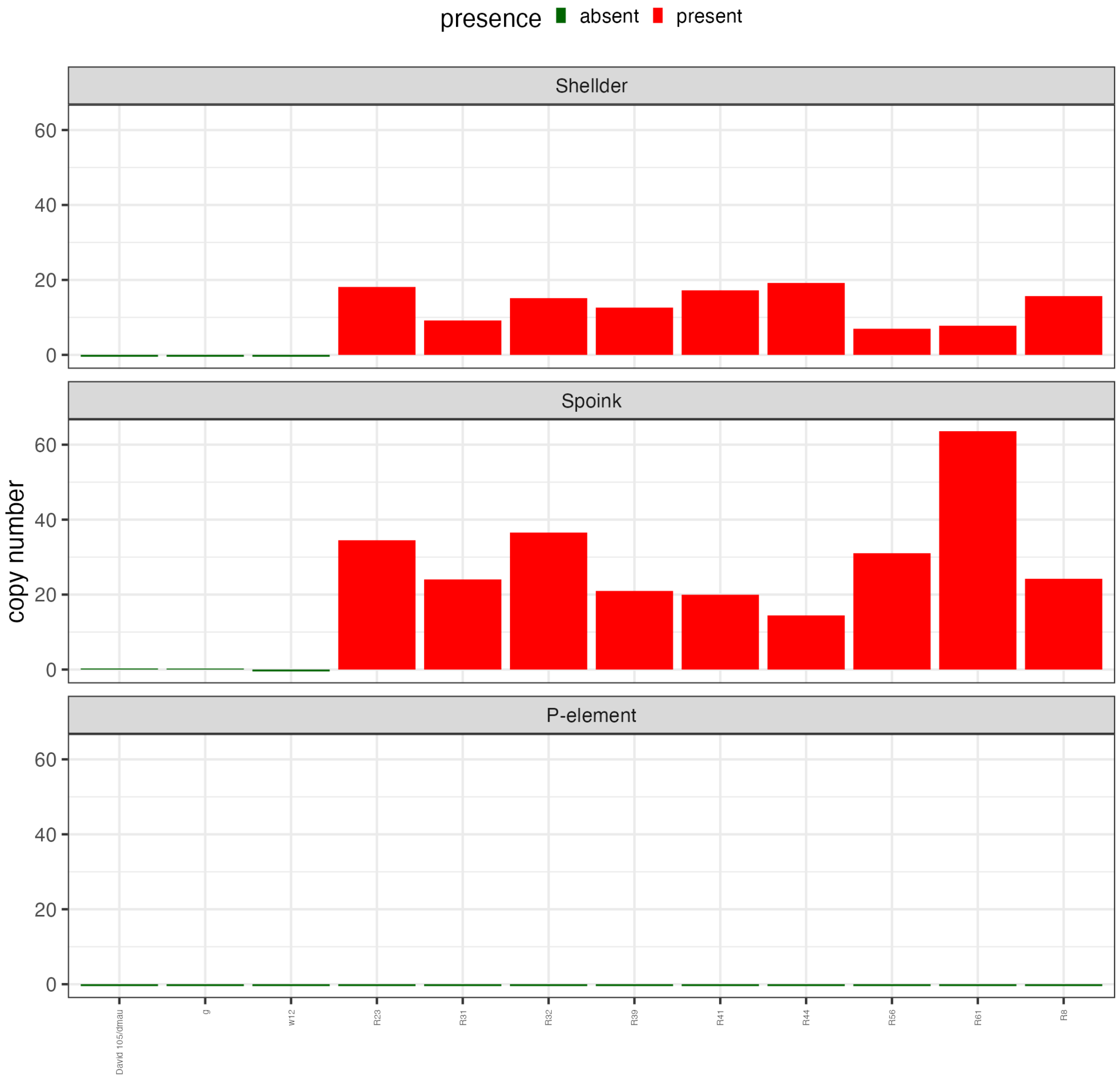

Figure 9: Abundance of *Spoink*, *Shellder* and the *P-element* insertions in 12 *D. mauritiana* strains collected during the last 50 years. Note that *Spoink* and *Shellder* are either both present or absent in a given strain.

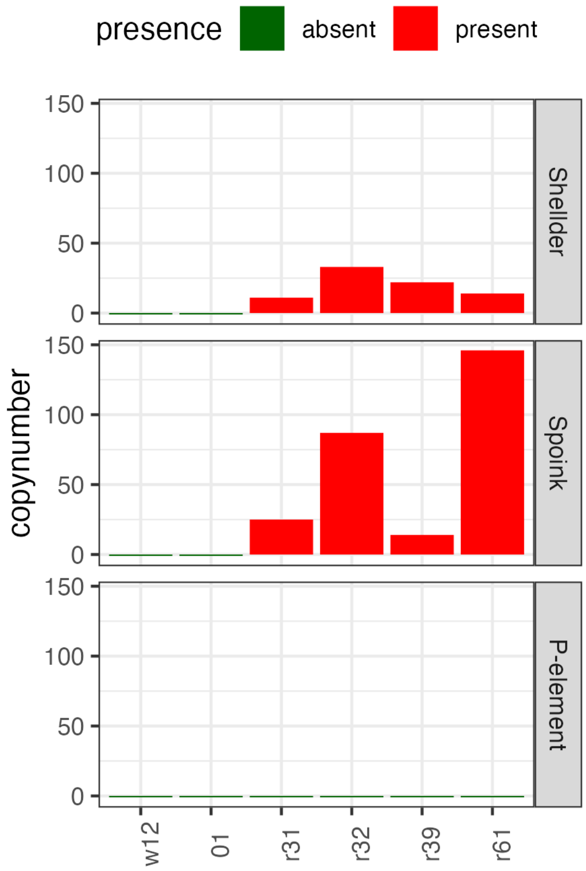

Figure 10: Abundance of *Shellder*, *Spoink* and *P-element* insertions in 5 long read assemblies of different *D. mauritiana* strains. Insertions were identified with RepeatMasker and hits with less than 750bp length or more than 10% divergence were removed. Strains are ordered by collection year.

.

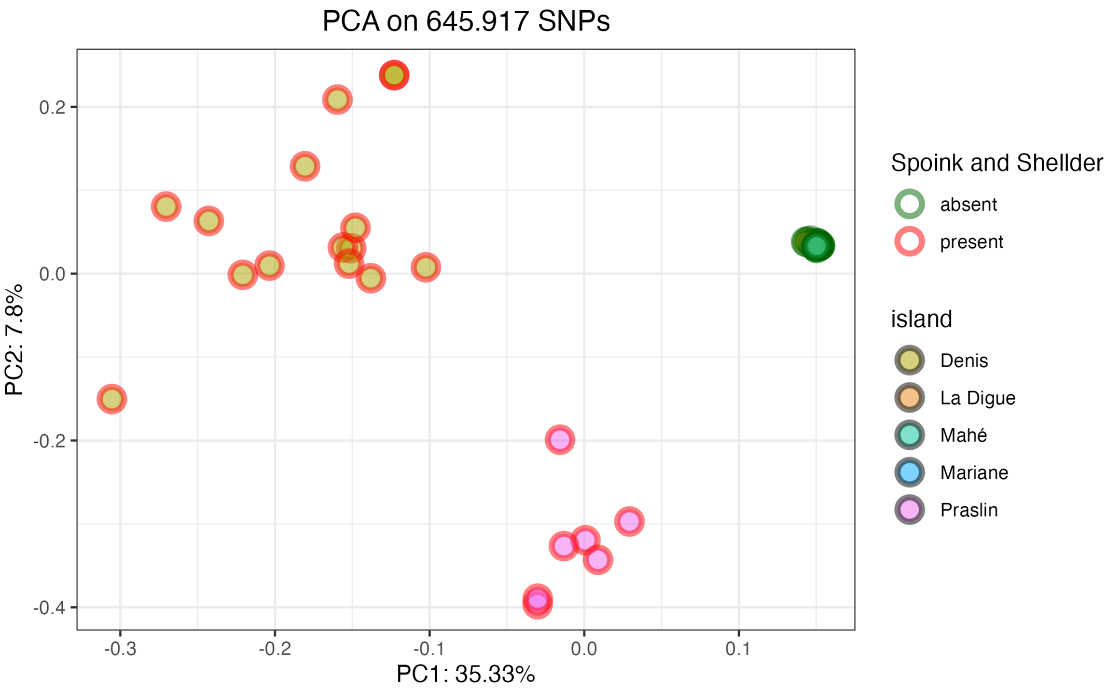

Figure 11: PCA based on more than 600.000 SNPs showing the relatedness among the different *D. sechellia* strains. Note that strains having *Spoink* and *Shellder* (green border) are highly related forming a separate group from strains not having the two TEs (red border).

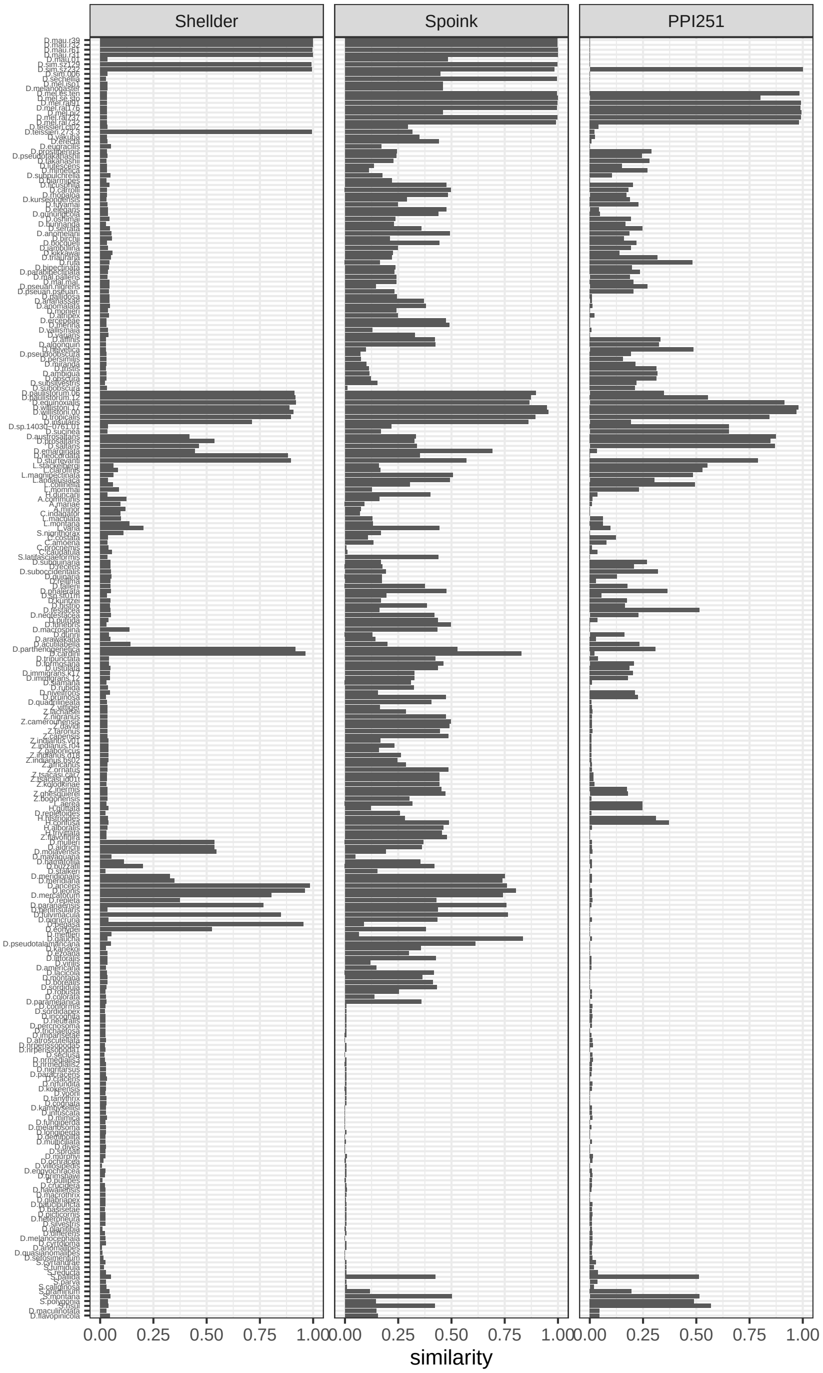

Figure 12: Similarity of TE insertions in long-read assemblies of 266 species with the consensus sequence of *Shellder*, *Spoink* and the *P-element*. In contrast to a similar figure in the main manuscript, the labels are provided in this figure, in order to enable identification of species of interest.

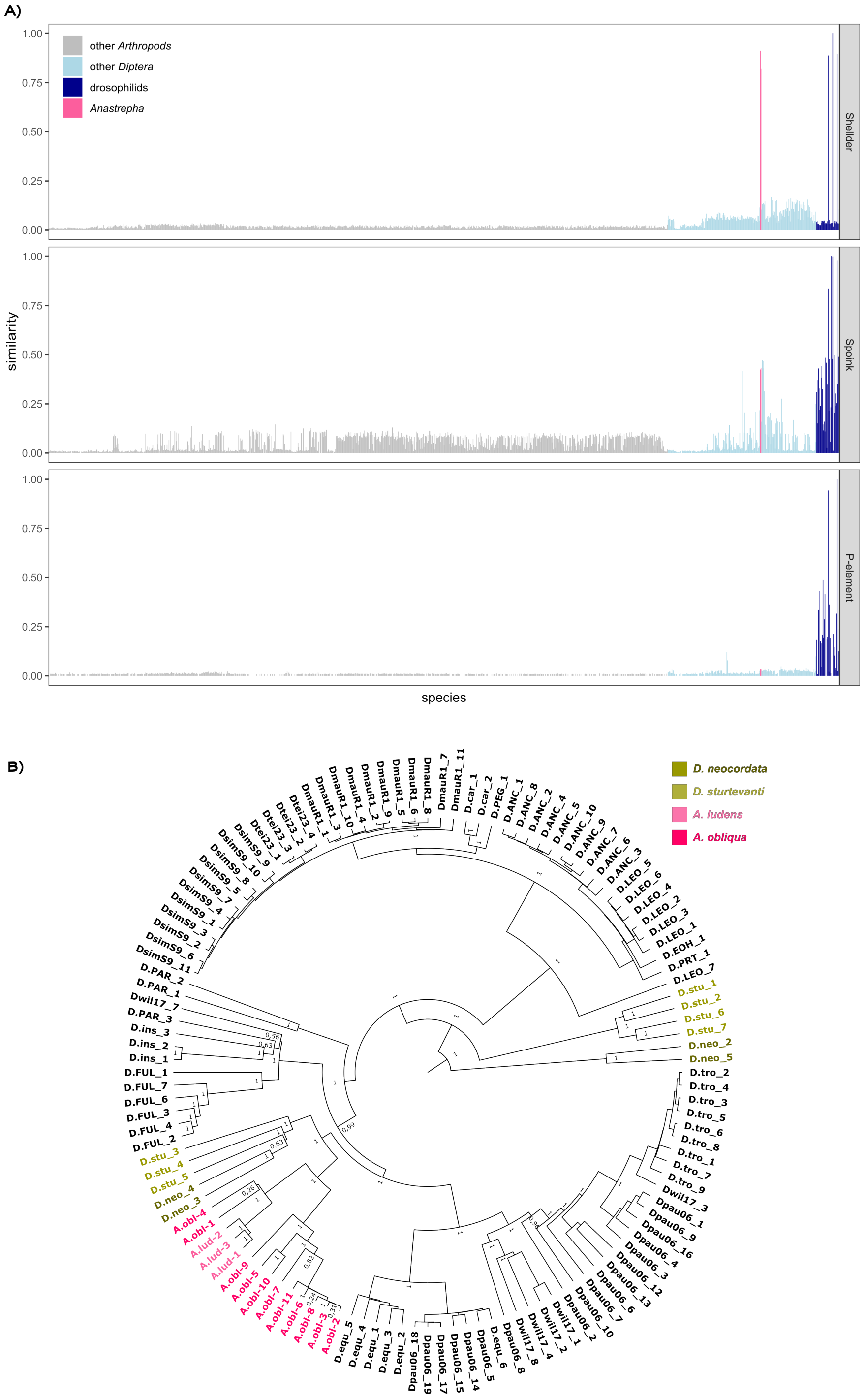

Figure 13: *Shellder* spread to *Anastrepha ludens* and *Anastrepha obliqua* likely following horizontal transfer from the *saltans* group. A) Similarity of the consensus sequence of *Shellder*, *Spoink* and the *P-element* with TE insertions in 1226 reference genomes of arthropods species. The barplots show for each species the similarity between the given TE and the best match in an assembly. For example a value of 0.9 indicates that at least one insertion in an assembly has a high similarity to the given TE. B) Bayesian tree of *Shellder* insertions in species having at least one full-length insertions (> 80% of length).

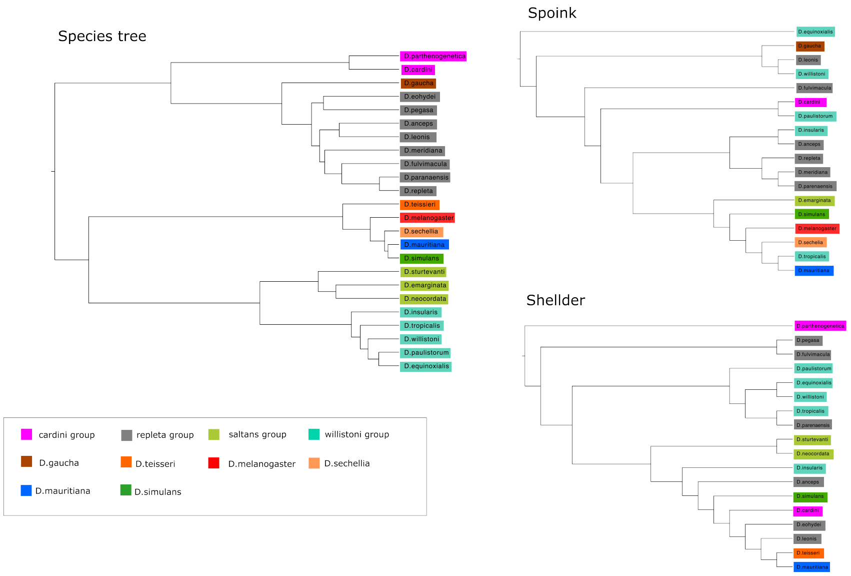

Figure 14: Discordance between the phylogenetic trees of *Spoink* and *Shellder* and the host species. A) Phyolgenetic tree of the species having full length insertions of either *Spoink* or *Shellder*. The tree is based on 393 BUSCO genes. B) Phylogenetic tree of *Spoink*. C) Phylogenetic tree of *Shellder*; for both TEs the trees were generated using the consensus sequences of each species. Only species having a full-length insertion are shown.

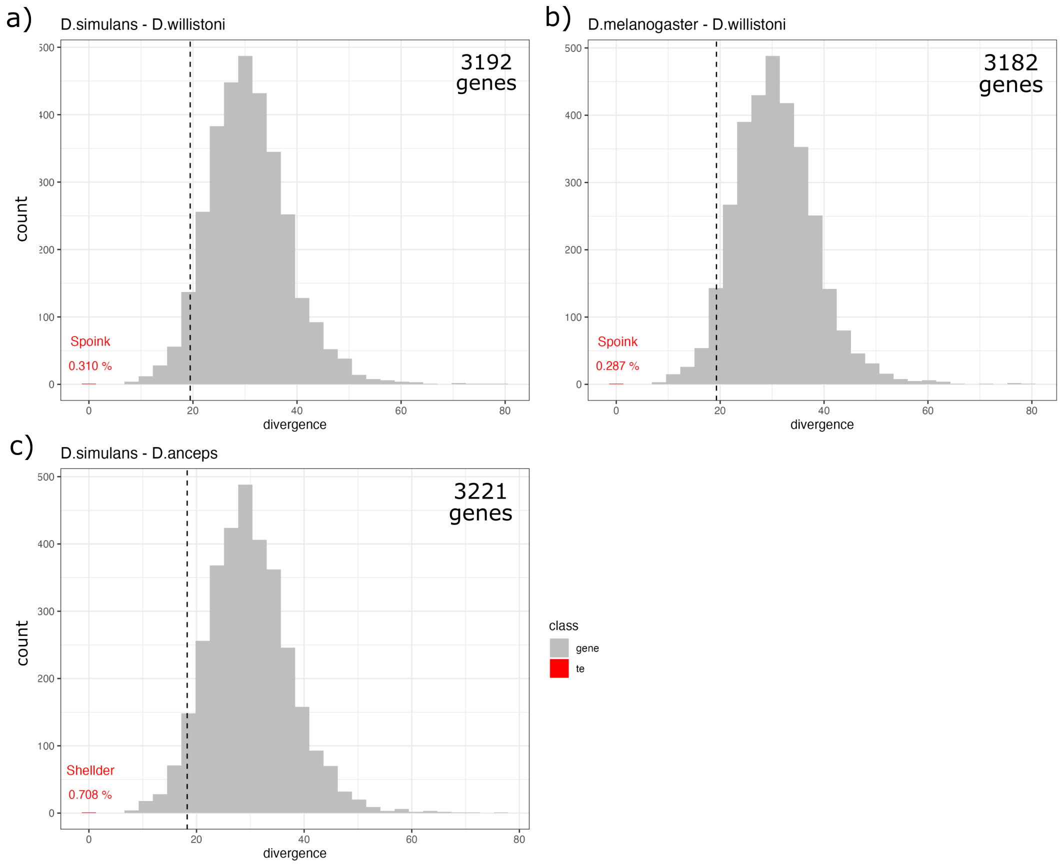

Figure 15: Divergence of the host genes compared to the divergence of *Spoink* (A, B) and *Shellder* (C). The distribution is shown for pairs of species that were likely involved in the horizontal transfer of both TEs. Dashed lines indicate the quantile of the 5% least diverged host genes. Note that the divergence of *Spoink* and *Shellder* (in percent; below TE name) is substantially lower than the divergence of any of the host genes, consistent with a horizontal transfer of the TE between the species. We used BUSCO [Manni et al., 2021] to extract the sequences of orthologous genes. Pairs of orthologous sequences were aligned with MUSCLE, and the divergence was computed from the alignment. For the TE we show the divergence between the consensus sequence (generated from insertions in *D. melanogaster* or *D. simulans*) and the best matching sequence identified by RepeatMasker (based on the highest alignment score, which favors full-length insertions).

Supplementary tables

Table 1: We discovered the recent invasions in *D. simulans* by noticing differences in the abundance of *Spoink* and *Gypsy_29_DWil_I_LTR_Gypsy* (from the repeat library of [Chakraborty et al., 2021]) between the reference genome *006* and a long-read assemblies from a more recently collected strain (*sz232*, collected in 2012 in Zuma Beach, CA [Signor et al., 2023] The best ten matches for both TEs are shown in each assembly. Matches were identified with RepeatMasker [Smit et al., 2013-2015].

score	subst	len	chrm	start	end	rc	TE	assembly
21804	19.80	0.84	JAEIGU010000232.1	265397	269681	C	Spoink	006
14038	20.79	0.58	JAEIGU010000170.1	20715966	20718946	C	Spoink	006
8036	32.52	0.76	JAEIGU010000154.1	516917	520853	C	Spoink	006
7927	30.00	0.62	JAEIGU010000028.1	1326184	1329499	+	Spoink	006
7917	32.54	0.76	JAEIGU010000028.1	718235	722171	C	Spoink	006
7650	31.89	0.70	JAEIGU010000251.1	2264076	2267697	+	Spoink	006
7462	30.10	0.62	JAEIGU010000028.1	1346416	1349732	+	Spoink	006
7224	29.68	0.52	JAEIGU010000170.1	20101699	20104435	C	Spoink	006
7197	29.72	0.52	JAEIGU010000170.1	20092583	20095319	C	Spoink	006
7155	29.68	0.52	JAEIGU010000170.1	20097140	20099879	C	Spoink	006
47859	0.17	1.00	JBBODM010000431.1	2360399	2365648	C	Spoink	sz232
43430	0.09	0.89	JBBODM010000444.1	12992636	12997303	+	Spoink	sz232
43392	0.06	0.89	JBBODM010000444.1	18858084	18862746	+	Spoink	sz232
43253	0.04	0.89	JBBODM010000510.1	180211	184877	+	Spoink	sz232
42991	0.13	0.89	JBBODM010000034.1	21052	25737	C	Spoink	sz232
42898	0.19	0.90	JBBODM010000430.1	1098403	1103116	+	Spoink	sz232
41391	0.25	0.87	JBBODM010000350.1	11151	15627	C	Spoink	sz232
40615	0.13	0.90	JBBODM010000495.1	810554	815223	+	Spoink	sz232
40107	0.21	0.84	JBBODM010000191.1	4617	8991	+	Spoink	sz232
270	16.07	0.01	JAEIGU010000201.1	16219591	16219646	C	Gypsy_29_DWil_I_LTR_Gypsy	006
263	27.03	0.01	JAEIGU010000170.1	18317597	18317670	C	Gypsy_29_DWil_I_LTR_Gypsy	006
244	20.59	0.01	JAEIGU010000044.1	20677868	20677935	+	Gypsy_29_DWil_I_LTR_Gypsy	006
238	16.42	0.01	JAEIGU010000201.1	7635521	7635587	C	Gypsy_29_DWil_I_LTR_Gypsy	006
236	12.77	0.01	JAEIGU010000044.1	12181606	12181652	C	Gypsy_29_DWil_I_LTR_Gypsy	006
236	14.04	0.01	JAEIGU010000044.1	8091055	8091111	+	Gypsy_29_DWil_I_LTR_Gypsy	006
236	23.08	0.01	JAEIGU010000044.1	18068828	18068892	C	Gypsy_29_DWil_I_LTR_Gypsy	006
233	23.81	0.01	JAEIGU010000086.1	11681488	11681550	+	Gypsy_29_DWil_I_LTR_Gypsy	006
232	15.38	0.01	JAEIGU010000044.1	5452554	5452605	+	Gypsy_29_DWil_I_LTR_Gypsy	006
225	22.25	0.01	JAEIGU010000028.1	1683211	1683275	C	Gypsy_29_DWil_I_LTR_Gypsy	006
48576	2.52	1.00	JBBODM010000525.1	450324	456077	+	Gypsy_29_DWil_I_LTR_Gypsy	sz232
48536	2.56	1.00	JBBODM010000510.1	2683380	2689137	C	Gypsy_29_DWil_I_LTR_Gypsy	sz232
48313	2.58	1.00	JBBODM010000494.1	305579	311329	C	Gypsy_29_DWil_I_LTR_Gypsy	sz232
48199	2.59	1.00	JBBODM010000465.1	222270	228021	+	Gypsy_29_DWil_I_LTR_Gypsy	sz232
45437	2.53	1.00	JBBODM010000566.1	21922	27665	C	Gypsy_29_DWil_I_LTR_Gypsy	sz232
45325	2.51	1.00	JBBODM010000291.1	432562	438314	C	Gypsy_29_DWil_I_LTR_Gypsy	sz232
44323	2.88	1.00	JBBODM010000292.1	5499	11242	C	Gypsy_29_DWil_I_LTR_Gypsy	sz232
264	16.07	0.01	JBBODM010000126.1	36882	36937	+	Gypsy_29_DWil_I_LTR_Gypsy	sz232
262	16.07	0.01	JBBODM010000467.1	1163445	1163500	+	Gypsy_29_DWil_I_LTR_Gypsy	sz232

Table 2: Overview of the 179 *D. melanogaster* short-read data analysed in this work. Data are from Grenier et al. [2015], Schwarz et al. [2021], Long et al. [2013], Lange et al. [2021], Rech et al. [2022], Shpak et al. [2023])

accession	strain	year	location
SRR23876586	museum	1933	Lund, Sweden
SRR11846555	Crimea	1936	Crimea, Ukraine
SRR11846565	Hikone-R	1958	Japan
SRR457698	A2(BOG1)	1962	Bogota, Colombia
SRR457707	A4(KSA2)	1963	South Africa
SRR457701	B4(RVC3)	1963	California, USA
SRR457669	A5(VAG1)	1965	Athens, Greece
SRR457697	A6(wild5B)	1966	Georgia, USA
SRR11846560	Harwich	1967	Harwich, MA, USA
SRR11460801	Pi2	1975	
SRR13257684	RI75-1	1975	Providence, USA
SRR14293191	RI75-2	1975	Providence, USA
SRR14293574	RI75-5	1975	Providence, USA
SRR14293140	RI75-10	1975	Providence, USA
SRR14293233	RI75-4	1975	Providence, USA
SRR14294393	RI75-7	1975	Providence, USA
SRR14294796	RI75-11	1975	Providence, USA
SRR14294940	RI75-13	1975	Providence, USA
SRR14293576	RI75-6	1975	Providence, USA
SRR14294899	RI75-12	1975	Providence, USA
SRR14294944	RI77-50	1977	Providence, USA
SRR14296646	RI77-52	1977	Providence, USA
SRR14296993	RI77-53	1977	Providence, USA
SRR14297433	RI77-61	1977	Providence, USA
SRR14297455	RI77-55	1977	Providence, USA
SRR14298094	RI77-60	1977	Providence, USA
SRR14296430	RI7751	1977	Providence, USA
SRR14297771	RI77-58	1977	Providence, USA
SRR14297795	RI77-56	1977	Providence, USA
SRR14298064	RI77-54	1977	Providence, USA
SRR14297441	RI78-1A	1978	Providence, USA
SRR14297770	RI78-6	1978	Providence, USA
SRR14297954	RI78-14	1978	Providence, USA
SRR14298028	RI78-1B	1978	Providence, USA
SRR14297434	RI78-12	1978	Providence, USA
SRR14297796	RI78-11	1978	Providence, USA
SRR14297813	RI78-8	1978	Providence, USA
SRR14297794	RI78-13	1978	Providence, USA
SRR14298002	RI78-5	1978	Providence, USA
SRR14298055	RI78-7	1978	Providence, USA
SRR14298091	RI78-9	1978	Providence, USA
SRR14297456	RI79-7	1979	Providence, USA
SRR14298093	RI79-3	1979	Providence, USA
SRR14306823	RI79-10	1979	Providence, USA
SRR14306826	RI79-17	1979	Providence, USA
SRR14306827	RI79-16	1979	Providence, USA
SRR14306825	RI79-19	1979	Providence, USA
SRR14306829	RI79-14	1979	Providence, USA
SRR14306830	RI79-13	1979	Providence, USA
SRR14298105	RI79-1	1979	Providence, USA
SRR14306822	RI79-20	1979	Providence, USA
SRR14306824	RI79-9	1979	Providence, USA
SRR14306828	RI79-15	1979	Providence, USA
SRR14306831	RI79-12	1979	Providence, USA
SRR14306842	RI79-11	1979	Providence, USA
SRR14306841	RI80-15	1980	Providence, USA
SRR14306844	RI80-11	1980	Providence, USA
SRR14306847	RI80-7	1980	Providence, USA
SRR14306850	RI80-4	1980	Providence, USA
SRR14306821	RI80-2	1980	Providence, USA
SRR14306843	RI80-13	1980	Providence, USA
SRR14306845	RI80-9	1980	Providence, USA
SRR14306846	RI80-8	1980	Providence, USA
SRR14306848	RI80-6	1980	Providence, USA
SRR14306849	RI80-5	1980	Providence, USA
SRR14306833	RI83-8	1983	Providence, USA
SRR14306835	RI83-6	1983	Providence, USA
SRR14306837	RI83-4	1983	Providence, USA
SRR14306838	RI83-3	1983	Providence, USA
SRR14306834	RI83-7	1983	Providence, USA
SRR14306832	RI83-9	1983	Providence, USA
SRR14306836	RI83-5	1983	Providence, USA
SRR14306839	RI83-2	1983	Providence, USA
SRR14306840	RI83-1	1983	Providence, USA
SRR1663598	ZH23	1993	Zimbabwe
SRR1663599	ZH26	1993	Zimbabwe
SRR1663600	ZH33	1993	Zimbabwe
SRR1663601	ZH42	1993	Zimbabwe
SRR1663602	ZS10	1993	Zimbabwe
SRR1663603	ZW09	1993	Zimbabwe
SRR1663604	ZW139	1993	Zimbabwe
SRR1663605	ZW140	1993	Zimbabwe
SRR1663606	ZW142	1993	Zimbabwe
SRR1663607	ZW144	1993	Zimbabwe
SRR1663608	ZW155	1993	Zimbabwe
SRR1663609	ZW177	1993	Zimbabwe
SRR1663610	ZW184	1993	Zimbabwe
SRR1663611	ZW185	1993	Zimbabwe
SRR1663530	B11	1995	Beijing, China
SRR1663531	B12	1995	Beijing, China
SRR1663532	B14	1995	Beijing, China
SRR1663533	B23	1995	Beijing, China
SRR1663534	B28	1995	Beijing, China
SRR1663535	B38	1995	Beijing, China
SRR1663536	B42	1995	Beijing, China
SRR1663537	B43	1995	Beijing, China
SRR1663538	B51	1995	Beijing, China
SRR1663539	B52	1995	Beijing, China
SRR1663540	B54	1995	Beijing, China
SRR1663541	B59	1995	Beijing, China
SRR1663561	N01	2003	Netherlands
SRR1663562	N02	2003	Netherlands
SRR1663563	N03	2003	Netherlands
SRR1663564	N04	2003	Netherlands
SRR1663565	N07	2003	Netherlands
SRR1663566	N10	2003	Netherlands
SRR1663567	N11	2003	Netherlands
SRR1663568	N13	2003	Netherlands
SRR1663569	N14	2003	Netherlands
SRR1663570	N15	2003	Netherlands
SRR1663571	N16	2003	Netherlands
SRR1663572	N17	2003	Netherlands
SRR1663573	N18	2003	Netherlands
SRR1663574	N19	2003	Netherlands
SRR1663575	N22	2003	Netherlands
SRR1663576	N23	2003	Netherlands
SRR1663577	N25	2003	Netherlands
SRR1663578	N29	2003	Netherlands
SRR1663579	N30	2003	Netherlands
SRR1663580	T01	2003	Tasmania, Australia
SRR1663581	T04	2003	Tasmania, Australia
SRR1663582	T05	2003	Tasmania, Australia
SRR1663583	T07	2003	Tasmania, Australia
SRR1663584	T09	2003	Tasmania, Australia
SRR1663585	T10 female	2003	Tasmania, Australia
SRR1663586	T14A	2003	Tasmania, Australia
SRR1663587	T22A	2003	Tasmania, Australia
SRR1663588	T23	2003	Tasmania, Australia
SRR1663589	T24	2003	Tasmania, Australia
SRR1663590	T25A	2003	Tasmania, Australia
SRR1663591	T29A	2003	Tasmania, Australia
SRR1663592	T30	2003	Tasmania, Australia
SRR1663593	T35	2003	Tasmania, Australia
SRR1663594	T36B	2003	Tasmania, Australia
SRR1663595	T39	2003	Tasmania, Australia
SRR1663596	T43A	2003	Tasmania, Australia
SRR1663597	T45B	2003	Tasmania, Australia
SRR1663542	I01	2004	Ithaca, USA
SRR1663543	I02	2004	Ithaca, USA
SRR1663544	I03	2004	Ithaca, USA
SRR1663545	I04	2004	Ithaca, USA
SRR1663546	I06	2004	Ithaca, USA
SRR1663547	I07	2004	Ithaca, USA
SRR1663548	I13	2004	Ithaca, USA
SRR1663549	I16	2004	Ithaca, USA
SRR1663550	I17	2004	Ithaca, USA
SRR1663551	I22	2004	Ithaca, USA
SRR1663552	I23	2004	Ithaca, USA
SRR1663553	I24	2004	Ithaca, USA
SRR1663554	I26	2004	Ithaca, USA
SRR1663555	I29	2004	Ithaca, USA
SRR1663556	I31	2004	Ithaca, USA
SRR1663557	I33	2004	Ithaca, USA
SRR1663558	I34	2004	Ithaca, USA
SRR1663559	I35	2004	Ithaca, USA
SRR1663560	I38	2004	Ithaca, USA
SRR14308759	spring4	2014	Providence, USA
SRR14308763	fall6	2014	Providence, USA
SRR14308767	spring12	2014	Providence, USA
SRR14308768	spring11	2014	Providence, USA
SRR14308772	spring7	2014	Providence, USA
SRR14308776	fall1	2014	Providence, USA
SRR14308762	spring1	2014	Providence, USA
SRR14308764	fall5	2014	Providence, USA
SRR14308766	fall3	2014	Providence, USA
SRR14308774	spring5	2014	Providence, USA
SRR14308775	fall2	2014	Providence, USA
SRR14308760	spring3	2014	Providence, USA
SRR14308761	spring2	2014	Providence, USA
SRR14308765	fall4	2014	Providence, USA
SRR14308769	spring10	2014	Providence, USA
SRR14308770	spring9	2014	Providence, USA
SRR14308771	spring8	2014	Providence, USA
SRR14308773	spring6	2014	Providence, USA
SRR9951090	ES_Ten_15_15	2015	Tenerife, Spain

Table 3: Overview of the 88 analysed *D. simulans* strains [Courret et al., 2023, Jackson et al., 2017, Sedghifar et al., 2016, Hill et al., 2016, Signor et al., 2018, Serrato-Capuchina et al., 2021, Wang et al., 2023].

**strain**	**SRR number**	**year**	**location**
w501	SRR12340887	1945	NA
w501	SRR2132976	1945	NA
Guy56	SRR22548179	1956	Guyana
14021-0251.006	SRR6425999	1961	NA
Cal61	SRR22548180	1961	California, USA
Ken75	SRR22548178	1975	Kenya
wxd1	SRR5491305	1980	NA
Sey81_rep2	SRR22548183	1981	Seychelles
Sey81_rep1	SRR22548195	1981	Seychelles
Tun83_rep1	SRR22548198	1983	Tunisia
Tun83_rep2	SRR22548184	1983	Tunisia
New91	SRR22548189	1991	New Caledonia
Fra93	SRR22548186	1993	France
Tan96	SRR22548196	1996	Tanzania
Zim97	SRR22548177	1997	Zimbabwe
Mad98	SRR22548187	1998	Madagascar
Sao01	SRR22548191	2001	Sao Tome
MD225a	ERR668322	2002	Madagascar
MD225b	ERR668323	2002	Madagascar
MD235	ERR668324	2002	Madagascar
MD72	ERR668317	2002	Madagascar
MD146	ERR668318	2002	Madagascar
MD201	ERR668320	2002	Madagascar
MD224	ERR668321	2002	Madagascar
MD238	ERR668325	2002	Madagascar
MD243	ERR668326	2002	Madagascar
MD255	ERR668327	2002	Madagascar
MD03	ERR668316	2002	Madagascar
MD197	ERR668319	2002	Madagascar
Sey03	SRR22548185	2003	Seychelles
QLD(AU)	SRR3091605	2004	Australia
TAS(AU)	SRR3091579	2004	Tasmania, Australia
NS64	ERR668310	2006	Kenya
NS111	ERR668314	2006	Kenya
NS116	ERR668315	2006	Kenya
NS49	ERR668308	2006	Kenya
NS95	ERR668312	2006	Kenya
NS96	ERR668313	2006	Kenya
NS11	ERR668305	2006	Kenya
NS19	ERR668306	2006	Kenya
NS37	ERR668307	2006	Kenya
NS63	ERR668309	2006	Kenya
NS89	ERR668311	2006	Kenya
Haw07	SRR22548188	2007	Hawaii
Egy08	SRR22548197	2008	Egypt
May09	SRR22548194	2009	Mayotte
Fra09	SRR22548193	2009	France
c211	ERR694697	2010	Florida, USA
basepop	ERR694694	2010	Florida, USA
c116	ERR694695	2010	Florida, USA
Aus11	SRR22548182	2011	Australia
FL(US)	SRR3084252	2011	Florida, USA
RI(US)	SRR3091655	2011	Rhode Island, USA
SZ232	SRR3585779	2012	California, USA
SZ244	SRR3585391	2012	California, USA
SZ45	SRR3585440	2012	California, USA
Mor12	SRR22548181	2012	Morocco
Egy12	SRR22548176	2012	Egypt
Cam16	SRR22548190	2016	Cameroon
Mor16	SRR22548192	2016	Morocco
18CHAALES15	SRR11456793	2018	Kenya
18KARI01	SRR11456782	2018	Kenya
18KARI03	SRR11456771	2018	Kenya
18KARI04	SRR11456760	2018	Kenya
18KARI05	SRR11456753	2018	Kenya
18KARI06	SRR11456752	2018	Kenya
18KARI09	SRR11456751	2018	Kenya
18KARI10	SRR11456750	2018	Kenya
18KARI26	SRR11456803	2018	Kenya
18KARI37	SRR11456802	2018	Kenya
18KARITANA20	SRR11456801	2018	Kenya
18MPALA02	SRR11456800	2018	Kenya
18MPALA02B	SRR11456799	2018	Kenya
18MPALA03	SRR11456797	2018	Kenya
18MPALA05	SRR11456796	2018	Kenya
18MPALA09	SRR11456794	2018	Kenya
18MPALA11	SRR11456792	2018	Kenya
18MPALA12	SRR11456791	2018	Kenya
18MPALA13	SRR11456790	2018	Kenya
18MU04	SRR11456789	2018	Senegal
18MU05	SRR11456788	2018	Senegal
18NANY04	SRR11456787	2018	Senegal
KARI25	SRR11456778	2018	Kenya
dsim_27_F0	ERR9452541	2020	Spain
dsim_31_F0	ERR9452754	2020	Spain
dsim_28_F0	ERR9439680	2020	Spain
dsim_29_F0	ERR9453536	2020	Spain
dsim_25_F0	ERR9436064	2020	Spain
dsim_26_F0	ERR9452532	2020	Spain

Table 4: Copy number of *Spoink*, *Shellder*, and *P-element* in long-read assemblies of *D. simulans*. TE insertions in the assemblies were identified with RepeatMasker. We required copies of *Shellder* and *Spoink* to have a length of at least 4 kb and to contain at least one LTR . An insertion is annotated as being present in *flamenco* if it is found in either of the two duplicates of *flamenco* in *D. simulans* [Signor et al., 2023]. If the locus is known to be incompletely assembled and no copy was found, it is listed as not applicable (NA).

**Strain**	**Collection**	***Shellder***		***Spoink***		***P-element***	
		**count**	**flamenco**	**count**	**flamenco**	**count**	**flamenco**
w^{501}	30-60s	0	0	0	0	0	0
14021.006	CA 1961	0	0	0	0	0	0
w^{xD1}	CA 80s	0	0	0	0	0	0
MD251	Mad. 2002	15	1	10	0	0	0
NS40	Kenya 2006	8	1	15	0	0	0
NS137	Kenya 2006	8	NA	10	NA	0	NA
SZ45	CA 2012	9	1	30	NA	28	NA
SZ129	CA 2012	11	1	8	NA	0	NA
SZ232	CA 2012	7	1	18	0	34	0
SZ244	CA 2012	6	NA	18	NA	0	NA
MD106	Mad. 2002	21	1	10	0	0	0
LNP-15-063	Zambia 2012	7	1	8	0	10	0
MD242	Mad. 2002	22	1	8	0	0	0

Table 5: Position of *Spoink* and *Shellder* insertions in the *D. simulans* assembly SZ129 ([Signor et al., 2023]).

**TE**	**contig**	**start**	**end**
Spoink	JBBODN010000001.1	4976343	4981554
Spoink	JBBODN010000001.1	25218020	25214211
Spoink	JBBODN010000010.1	5679564	5683582
Spoink	JBBODN010000189.1	9151482	9147526
Spoink	JBBODN010000189.1	4322055	4318243
Spoink	JBBODN010000189.1	1809622	1805816
Spoink	JBBODN010000014.1	3009534	3013345
Spoink	JBBODN010000030.1	27295	30893
Shellder	JBBODN010000154.1	6650	13278
Shellder	JBBODN010000201.1	28165	21542
Shellder	JBBODN010000014.1	13905210	13911837
Shellder	JBBODN010000188.1	227114	233746
Shellder	JBBODN010000029.1	273808	267187
Shellder	JBBODN010000122.1	31720	25089
Shellder	JBBODN010000186.1	113208	106592
Shellder	JBBODN010000185.1	11331866	11325262
Shellder	JBBODN010000038.1	56552	51971
Shellder	JBBODN010000032.1	284801	280699
Shellder	JBBODN010000032.1	370173	373922

Table 6: Overview of the 12 analysed *D. mauritiana* strains [Miller et al., 2018, Sproul et al., 2020, Lee and Karpen, 2017, Garrigan et al., 2012].

**strain**	**SRR number**	**year**
David 105/dmau	SRR6425993	1970
g	SRR10728582	1979
w12	SRR19973828	1980
R31	SRR1560095	2006
R41	SRR1560132	2006
R44	SRR1560147	2006
R56	SRR1560148	2006
R23	SRR1560087	2006
R32	SRR1560103	2006
R61	SRR1560268	2006
R8	SRR1560275	2006
R39	SRR1560108	2006

Table 7: Position of *Spoink* and *Shellder* insertions in the *D. mauritiana* assembly R31.

**TE**	**contig**	**start**	**end**
Spoink	JBAMBX010000094.1	20426544	20431765
Spoink	JBAMBX010000322.1	1373321	1378544
Spoink	JBAMBX010000322.1	1291426	1296095
Spoink	JBAMBX010000318.1	126680	121472
Spoink	JBAMBX010000019.1	18135584	18130913
Spoink	JBAMBX010000019.1	8666057	8661543
Spoink	JBAMBX010000398.1	19093600	19098271
Spoink	JBAMBX010000002.1	1659752	1655238
Spoink	JBAMBX010000002.1	5533660	5538174
Spoink	JBAMBX010000002.1	2347572	2352086
Spoink	JBAMBX010000066.1	224475	228989
Spoink	JBAMBX010000400.1	417116	412602
Spoink	JBAMBX010000400.1	825113	829627
Spoink	JBAMBX010000074.1	2107776	2112290
Spoink	JBAMBX010000069.1	55839	51325
Spoink	JBAMBX010000015.1	294192	289678
Spoink	JBAMBX010000475.1	4681	165
Spoink	JBAMBX010000323.1	19991	24505
Spoink	JBAMBX010000204.1	131010	135524
Spoink	JBAMBX010000023.1	6083792	6087848
Shelleder	JBAMBX010000019.1	2703552	2710191
Shelleder	JBAMBX010000019.1	10431634	10424995
Shelleder	JBAMBX010000019.1	4194402	4201039
Shelleder	JBAMBX010000094.1	16639055	16632416
Shelleder	JBAMBX010000191.1	444336	450975
Shelleder	JBAMBX010000400.1	1348597	1341958
Shelleder	JBAMBX010000074.1	1734462	1741101
Shelleder	JBAMBX010000198.1	157459	164096
Shelleder	JBAMBX010000023.1	10679150	10672511
Shelleder	JBAMBX010000134.1	10	6644

Table 8: Overview of the 43 analysed *D. sechellia* short-reads data [Matute and Ayroles, 2014, Schrider et al., 2018].

**strain**	**SRR number**	**year**	**location**
Denis72	SRR14138506	2012	Denis
Anro71	SRR14138507	2012	Mahé
maria_3	SRR5860570	2012	Mariane
Anro_B8	SRR5860573	2012	Mahé
LD8	SRR5860582	2012	La Digue
PNF4	SRR5860583	2012	Praslin
LD15	SRR5860584	2012	La Digue
DenisNF155	SRR5860625	2012	Denis
DenisNoni10	SRR5860626	2012	Denis
DenisDNJ6	SRR5860627	2012	Denis
DenisNoni60	SRR5860628	2012	Denis
DenisAT3	SRR5860629	2012	Denis
DenisJT1	SRR5860630	2012	Denis
Denis124	SRR5860631	2012	Denis
Denis7_2	SRR5860632	2012	Denis
DenisNF13	SRR5860633	2012	Denis
DenisNF66	SRR5860634	2012	Denis
DenisNF100	SRR5860639	2012	Denis
DenisNF123	SRR5860640	2012	Denis
DenisAMT	SRR5860643	2012	Denis
DenisNF134	SRR5860644	2012	Denis
Anro_B7	SRR5860645	2012	Mahé
LD16	SRR5860656	2012	La Digue
LD13	SRR5860657	2012	La Digue
LD14	SRR5860658	2012	La Digue
mariane_1	SRR5860659	2012	Mariane
Anro_B1	SRR5860660	2012	Mahé
Anro_B2	SRR5860661	2012	Mahé
LD12	SRR5860662	2012	La Digue
Anro_B6	SRR5860663	2012	Mahé
LD11_sech	SRR5860664	2012	La Digue
Anro_B3	SRR5860665	2012	Mahé
PNF10	SRR5860666	2012	Praslin
PNF11	SRR5860667	2012	Praslin
DenisNoni101	SRR5860668	2012	Denis
Denis7_8	SRR5860669	2012	Denis
DenisMCL	SRR5860670	2012	Denis
Denis135	SRR5860671	2012	Denis
PNF8	SRR5860672	2012	Praslin
PNF5	SRR5860673	2012	Praslin
PNF3	SRR5860674	2012	Praslin
PNF7	SRR5860675	2012	Praslin
Anro_B5	SRR5860676	2012	Mahé

Table 9: Overview of the 13 analysed *D. teissieri* short-reads data [Turissini and Matute, 2017, Cooper et al., 2018].

**strain**	**SRR number**	**location**
Balancha_1	SRR5860615	Bioko
Bata2	SRR5860576	Equatorial Guinea
Bata8	SRR5860577	Equatorial Guinea
cascade_2_1	SRR5860623	Bioko
cascade_2_2	SRR5860616	Bioko
cascade_2_4	SRR5860622	Bioko
cascade_4_1	SRR5860618	Bioko
cascade_4_2	SRR5860572	Bioko
cascade_4_3	SRR5860617	Bioko
House_Bioko	SRR5860621	Bioko
La_Lope_Gabon	SRR5860571	Gabon
Selinda	SRR5860620	Zimbabwe
Zimbabwe	SRR5860619	Zimbabwe

Table 10: Overview of the 38 analysed *D. yakuba* short-reads data [Turissini and Matute, 2017, Cooper et al., 2018, Yassin et al., 2016]

**strain**	**SRR number**	**location**
1_19	SRR5860601	São Tomé - hybrid zone
1_5	SRR5860600	São Tomé - hybrid zone
1_6	SRR5860598	São Tomé - hybrid zone
1_7	SRR5860649	São Tomé - hybrid zone
2_11	SRR5860596	São Tomé - hybrid zone
2_14	SRR5860599	São Tomé - hybrid zone
2_6	SRR5860593	São Tomé - hybrid zone
2_8	SRR5860603	São Tomé - hybrid zone
3_16	SRR5860602	São Tomé - hybrid zone
3_2	SRR5860590	São Tomé - hybrid zone
3_23	SRR5860655	São Tomé - hybrid zone
4_21	SRR5860647	São Tomé - hybrid zone
Abidjan_12	SRR5860586	Ivory Coast
Airport_16_5	SRR5860591	São Tomé - lowlands
Anton_1_Principe	SRR5860651	Príncipe
Anton_2_Principe	SRR5860585	Príncipe
BAR_1000_2	SRR5860579	São Tomé - hybrid zone
BIOKO_NE_4_6	SRR5860595	Bioko
Bosu_1235_14	SRR5860646	São Tomé - hybrid zone
Cascade_18	SRR5860592	São Tomé - lowlands
Cascade_19_16	SRR5860604	Bioko
Cascade_21	SRR5860578	Bioko
Cascade_SN6_1	SRR5860588	São Tomé - hybrid zone
COST_1235_2	SRR5860650	São Tomé - hybrid zone
COST_1235_3	SRR5860597	São Tomé - hybrid zone
Montecafe_17_17	SRR5860587	São Tomé - hybrid zone
OBAT_1200_5	SRR5860589	São Tomé - hybrid zone
SA_3	SRR5860652	São Tomé - hybrid zone
SanTome_city_14_26	SRR5860574	São Tomé - lowlands
SJ_1	SRR5860575	São Tomé - lowlands
SJ14	SRR5860653	São Tomé - lowlands
SJ4	SRR5860654	São Tomé - lowlands
SJ7	SRR5860594	São Tomé - lowlands
SN_Cascade_22	SRR5860581	São Tomé - hybrid zone
SN7	SRR5860580	São Tomé - hybrid zone
Tai_18	SRR5860648	Ivory Coast
yakAK	SRR2318687	Mayotte
yakLV	SRR2318706	Mayotte

Table 11: Overview of the analysed *D. santomea* [Matute and Ayroles, 2014], *D. erecta* [Yassin et al., 2016] and *D. orena* [Comeault et al., 2017] short-reads data.

**species strain**	**SRR number**	**location**	
D. santomea	BS14	SRR5860641	São Tomé
D. santomea	C550_39	SRR5860642	São Tomé
D. santomea	C650_14	SRR5860635	São Tomé
D. santomea	CAR1600	SRR5860637	São Tomé
D. santomea	Qiuja630.39	SRR5860605	São Tomé
D. santomea	Quija37	SRR5860610	São Tomé
D. santomea	Rain42	SRR5860636	São Tomé
D. santomea	san_Field3	SRR5860638	São Tomé
D. santomea	sanC1350.14	SRR5860606	São Tomé
D. santomea	sanCAR1490.5	SRR5860607	São Tomé
D. santomea	sanCOST1250.5	SRR5860624	São Tomé
D. santomea	sanCOST1270.6	SRR5860609	São Tomé
D. santomea	sanOBAT1200.13	SRR5860612	São Tomé
D. santomea	sanOBAT1200.5	SRR5860614	São Tomé
D. santomea	sanRain39	SRR5860613	São Tomé
D. santomea	sanSTO7	SRR5860611	São Tomé
D. santomea	sanThena5	SRR5860608	São Tomé
D. erecta	NN	SRR1977589	Gabon
D. erecta	N2	SRR1977539	cross
D. erecta	BC7	SRR1977582	cross
D. erecta	C3	SRR1977503	Gabon
D. orena	orena_bioko1	SRR5382770	Bioko

Table 12: Overview of *Shellder*, *Spoink* and *P-element* insertions in strains from different species of the *melanogaster* subgroup. For all available strains (N) we provide the number of strains having a given TE (positive) and the average copy number in those strains having the TE (mean). Copy number estimates were obtained with DeviaTE.

**Species**	**N**	***Shellder***		***Spoink***		***P-element***	
		**positive**	**mean**	**positive**	**mean**	**positive**	**mean**
D. melanogaster	183	0	0	105	21	171	17
D. simulans	88	66	11	66	14	33	13
D. sechellia	43	22	9	22	22	0	0
D. mauritiana	12	9	14	9	30	0	0
D. teissieri	13	8	13	0	0	0	0
D. yakuba	38	0	0	0	0	0	0
D. santomea	17	0	0	0	0	0	0
D. erecta	4	0	0	0	0	0	0
D. orena	1	0	0	0	0	0	0

Table 13: Segregating *Shellder* SNPs shared between species. A high number of shared SNPs between the pairs: *D. simulans*-*D. sechellia* and *D. simulans*-*D. mauritiana*, but not between *D. sechellia*-*D. mauritiana*, suggest two independent events of horizontal transfer from *D. simulans* to the two sister species.

**Species**	*D. sechellia*	*D. mauritiana*	*D. teissieri*
D. simulans	6	7	0
D. sechellia		1	0
D. mauritiana			0

References

M. Chakraborty, C. Chang, D. Khost, J. A. J Vedanayagam, Y. Liao, K. Montooth, C. Meiklejohn, A. Larracuente, and J. Emerson. Evolution of genome structure in the *Drosophila simulans* species complex. *Genome Research*, 31:380–396, 2021.

A. A. Comeault, A. Serrato-Capuchina, D. A. Turissini, P. J. McLaughlin, J. R. David, and D. R. Matute. A nonrandom subset of olfactory genes is associated with host preference in the fruit fly d*Drosophila orena*. *Evolution Letters*, 1(2):73–85, 2017.

B. S. Cooper, A. Sedghifar, W. T. Nash, A. A. Comeault, and D. R. Matute. A maladaptive combination of traits contributes to the maintenance of a *Drosophila* hybrid zone. *Current Biology*, 28(18):2940–2947, 2018.

C. Courret, D. Ogereau, C. Gilbert, A. M. Larracuente, and C. Montchamp-Moreau. The evolutionary history of *Drosophila simulans* y chromosomes reveals molecular signatures of resistance to sex ratio meiotic drive. *Molecular Biology and Evolution*, 40(7):msad152, 2023.

D. Garrigan, S. B. Kingan, A. J. Geneva, P. Andolfatto, A. G. Clark, K. R. Thornton, and D. C. Presgraves. Genome sequencing reveals complex speciation in the *Drosophila simulans* clade. *Genome research*, 22(8): 1499–1511, 2012.

L. S. Gramates, J. Agapite, H. Attrill, B. R. Calvi, M. A. Crosby, G. Dos Santos, J. L. Goodman, D. Goutte-Gattat, V. K. Jenkins, T. Kaufman, et al. Flybase: a guided tour of highlighted features. *Genetics*, 220 (4):iyac035, 2022.

J. K. Grenier, J. R. Arguello, M. C. Moreira, S. Gottipati, J. Mohammed, S. R. Hackett, R. Boughton, A. J. Greenberg, and A. G. Clark. Global diversity lines–a five-continent reference panel of sequenced *Drosophila melanogaster* strains. *G3: Genes, Genomes, Genetics*, 5(4):593–603, 2015.

T. Hill, C. Schlötterer, and A. J. Betancourt. Hybrid dysgenesis in *Drosophila simulans* associated with a rapid invasion of the p-element. *PLoS Genet*, 12(3):e1005920, 2016.

B. C. Jackson, J. L. Campos, P. R. Haddrill, B. Charlesworth, and K. Zeng. Variation in the intensity of selection on codon bias over time causes contrasting patterns of base composition evolution in *Drosophila*. *Genome Biology and Evolution*, 9(1):102–123, 2017.

V. V. Kapitonov and J. Jurka. Molecular paleontology of transposable elements in the *Drosophila melanogaster* genome. *Proceedings of the National Academy of Sciences of the United States of America*, 100(11):6569–74, 2003. ISSN 0027-8424.

J. D. Lange, H. Bastide, J. B. Lack, and J. E. Pool. A Population Genomic Assessment of Three Decades of Evolution in a Natural *Drosophila* Population. *Molecular Biology and Evolution*, 39(2), 2021.

Y. C. G. Lee and G. H. Karpen. Pervasive epigenetic effects of *Drosophila* euchromatic transposable elements impact their evolution. *Elife*, 6:e25762, 2017.

Q. Long, F. A. Rabanal, D. Meng, C. D. Huber, A. Farlow, A. Platzer, Q. Zhang, B. J. Vilhjálmsson, A. Korte, V. Nizhynska, et al. Massive genomic variation and strong selection in *Arabidopsis thaliana* lines from Sweden. *Nature genetics*, 45(8):884–890, 2013.

M. Manni, M. R. Berkeley, M. Seppey, and E. M. Zdobnov. Busco: assessing genomic data quality and beyond. *Current Protocols*, 1(12):e323, 2021.

D. Matute and J. Ayroles. Hybridization occurs between *Drosophila* simulans and *D. sechellia* in the seychelles archipelago. *Journal of evolutionary biology*, 27(6):1057–1068, 2014.

D. E. Miller, C. Staber, J. Zeitlinger, and R. S. Hawley. Highly Contiguous Genome Assemblies of 15 *Drosophila* Species Generated Using Nanopore Sequencing . *G3: Genes—Genomes—Genetics*, 8(10): 3131–3141, 2018.

R. Pianezza, A. Scarpa, P. Narayanan, S. Signor, and R. Kofler. Spoink, a ltr retrotransposon, invaded *D. melanogaster* populations in the 1990s. *bioRxiv*, 2023.

G. E. Rech, S. Radío, S. Guirao-Rico, L. Aguilera, V. Horvath, L. Green, H. Lindstadt, V. Jamilloux, H. Quesneville, and J. González. Population-scale long-read sequencing uncovers transposable elements associated with gene expression variation and adaptive signatures in *Drosophila. Nature Communications*, 13(1):1948, 2022.

D. R. Schrider, J. Ayroles, D. R. Matute, and A. D. Kern. Supervised machine learning reveals introgressed loci in the genomes of *Drosophila simulans* and *D. sechellia. PLoS Genet.*, 14(4):e1007341, Apr. 2018.

F. Schwarz, F. Wierzbicki, K.-A. Senti, and R. Kofler. Tirant Stealthily Invaded Natural *Drosophila melanogaster* Populations during the Last Century . *Molecular Biology and Evolution*, 38(4):1482–1497, 2021.

A. Sedghifar, P. Saelao, and D. J. Begun. Genomic patterns of geographic differentiation in *Drosophila simulans. Genetics*, 202(3):1229–1240, 2016.

A. Serrato-Capuchina, E. R. D'Agostino, D. Peede, B. Roy, K. Isbell, J. Wang, and D. R. Matute. P-elements strengthen reproductive isolation within the *Drosophila simulans* species complex. *Evolution*, 75 (10):2425–2440, 2021.

M. Shpak, H. R. Ghanavi, J. D. Lange, J. E. Pool, and M. C. Stensmyr. Genomes from historical drosophila melanogaster specimens illuminate adaptive and demographic changes across more than 200 years of evolution. *PLOS Biology*, 21(10):1–31, 10 2023. doi: 10.1371/journal.pbio.3002333. URL `https://doi.org/10.1371/journal.pbio.3002333`.

A. Shumate and S. Salzberg. Liftoff: accurate mapping of gene annotations. *Bioinformatics*, 37(12):1639–1643, 2021.

S. Signor, J. Vedanayagam, B. Kim, F. Wierzbicki, R. Kofler, and E. Lai. Rapid evolutionary diversification of the flamenco locus across simulans clade *Drosophila* species. *PLoS Genet*, 19, 2023.

S. A. Signor, F. N. New, and S. Nuzhdin. A large panel of *Drosophila simulans* reveals an abundance of common variants. *Genome biology and evolution*, 10(1):189–206, 2018.

A. F. A. Smit, R. Hubley, and P. Green. RepeatMasker Open-4.0, 2013-2015. URL `http://www.repeatmasker.org`.

J. S. Sproul, D. E. Khost, D. G. Eickbush, S. Negm, X. Wei, I. Wong, and A. M. Larracuente. Dynamic evolution of euchromatic satellites on the x chromosome in *Drosophila melanogaster* and the simulans clade. *Molecular biology and evolution*, 37(8):2241–2256, 2020.

D. A. Turissini and D. R. Matute. Fine scale mapping of genomic introgressions within the *Drosophila yakuba* clade. *PLoS genetics*, 13(9):e1006971, 2017.

Y. Wang, P. McNeil, R. Abdulazeez, M. Pascual, S. E. Johnston, P. D. Keightley, and D. J. Obbard. Variation in mutation, recombination, and transposition rates in *Drosophila melanogaster* and *Drosophila simulans. Genome Research*, 33(4):587–598, 2023.

A. Yassin, V. Debat, H. Bastide, N. Gidaszewski, J. R. David, and J. E. Pool. Recurrent specialization on a toxic fruit in an island *Drosophila population. Proceedings of the National Academy of Sciences*, 113(17): 4771–4776, 2016.
